# Effect of antiviral therapy on the outcomes of mechanically ventilated patients with herpes simplex virus detected in the respiratory tract: a systematic review and meta-analysis

**DOI:** 10.1186/s13054-020-03296-5

**Published:** 2020-09-29

**Authors:** Stefan Hagel, André Scherag, Lukas Schuierer, Reinhard Hoffmann, Charles-Edouard Luyt, Mathias W. Pletz, Miriam Kesselmeier, Sebastian Weis

**Affiliations:** 1 Institute for Infectious Diseases and Infection Control, Jena University Hospital – Friedrich Schiller University Jena, Jena, Germany; 2 Research Group Clinical Epidemiology, Center for Sepsis Control and Care (CSCC), Jena University Hospital – Friedrich Schiller University Jena, Jena, Germany; 3 Institute of Medical Statistics, Computer and Data Sciences, Jena University Hospital – Friedrich Schiller University Jena, Jena, Germany; 4 TUM Graduate School, Technical University of Munich (TUM), Munich, Germany; 5 Institute for Laboratory Medicine and Microbiology, University Hospital Augsburg, Augsburg, Germany; 6 Service de Médecine Intensive Réanimation, Institut de Cardiologie, Groupe Hospitalier Pitié–Salpêtrière, Sorbonne-Université, Assistance Publique Hôpitaux de Paris, Paris, France; 7 Department of Anesthesiology and Intensive Care Therapy, Jena University Hospital – Friedrich Schiller University Jena, Jena, Germany; 8 Center for Sepsis Control and Care, Jena University Hospital – Friedrich Schiller University Jena, Jena, Germany

**Keywords:** Herpes simplex, Mechanical ventilation, Antiviral therapy, Critically ill

## Abstract

**Background:**

Herpes simplex virus (HSV) is frequently detected in the respiratory tract of mechanically ventilated patients. The aim of this study was to assess current evidence to determine whether antiviral therapy is associated with better outcomes in these patients.

**Methods:**

*MEDLINE*, *ISI Web of Science*, *Cochrane Database* and ClinicalTrials.gov were searched from inception to 25 May 2020. All clinical studies investigating the effects of antiviral therapy on the outcome of mechanically ventilated ICU patients in whom HSV was detected in the respiratory tract were eligible for inclusion, regardless of study design, publication status or language. Titles and abstracts were reviewed independently by two authors. If the articles seemed eligible, full-text articles were reviewed and data extracted.

We performed a random-effects meta-analysis to estimate relative risks (RRs) with corresponding 95% confidence intervals (CIs). The primary endpoint was hospital all-cause mortality.

**Results:**

Nine studies were included in the meta-analysis (one randomized controlled trial, eight cohort studies). Antiviral treatment was associated with lower hospital mortality (with antiviral treatment, 40.6% (189 out of 465 patients); without, 52.7% (193 out of 366 patients); RR 0.74 [0.64, 0.85]; eight studies, low quality of evidence). Furthermore, antiviral treatment was associated with lower 30-day mortality (RR 0.75 [0.59, 0.94]; three studies, very low quality of evidence). We did not observe evidence for differences in ICU mortality (RR 0.73 [0.51, 1.05]; three studies, very low quality of evidence).

**Conclusions:**

This meta-analysis of the available data shows that antiviral therapy might result in lower hospital and 30-day all-cause mortality in mechanically ventilated ICU patients who are positive for HSV in the respiratory tract. However, this result must be interpreted with great caution due to the high risk of bias and limited number of patients. Large, well-designed randomized controlled clinical trials are urgently needed.

**Trial registration:**

The study was registered in advance on International Prospective Register of Systematic Reviews (CRD42020180053).

## Introduction

Herpes simplex virus (HSV) causes a variety of infections that affect mucocutaneous surfaces, the central nervous system and, occasionally, visceral organs. After primary infection, HSV invades neurons and subsequently remains in a non-replicating form in the sensory ganglia for the entire lifespan of the infected individual [1]. During the latency period, reactivation of the infection can be triggered by a wide range of stimuli, including local (e.g. tissue lesions or UV light) or systemic stimuli (e.g. fever, impairment of the immune system during critical illness or surgery) [1]. Oropharyngeal HSV reactivation has been shown to occur in 20 to 54% of critically ill patients, depending on the study population and the severity of disease [2–6]. In patients with prolonged mechanical ventilation, HSV can be detected in the bronchoalveolar lavage (BAL) in up to 64% of patients in intensive care units (ICUs) [7]. However, whether antiviral therapy improves patients’ outcomes in these circumstances is a matter of debate [8, 9]. It remains unclear whether the detection of HSV represents harmless viral shedding as a consequence of reactivation, reflecting the severity of the underlying disease and/or a surrogate for a state of decreased virological immune response (immunoparalysis), or a true clinical infection requiring antiviral therapy [9–11]. Study results are conflicting. Mortality in patients with HSV in respiratory secretions was increased in some [6, 12–14] but not in other studies [5, 7, 15]. The decision is further complicated by the challenge of rendering a confident clinical diagnosis of HSV bronchopulmonary infection in these patients [16]. The aim of the present systematic review and meta-analysis is to assess the current evidence as to whether antiviral therapy is associated with better outcomes in mechanically ventilated ICU patients in whom HSV was detected in the respiratory tract.

## Material and methods

The systematic review and meta-analysis were performed according to the Preferred Reporting Items for Systematic Reviews and Meta-Analyses (PRISMA) and Meta-Analysis of Observational Studies in Epidemiology (MOOSE) guidelines. Complete details, including electronic search strategy, objectives, criteria for study selection, eligibility, data collection and assessment of study quality, were registered in advance in the PROSPERO International Prospective Register of Systematic Reviews (CRD42020180053).

### Literature search and data extraction

All clinical studies investigating the effects of antiviral therapy on the outcome of mechanically ventilated ICU patients in whom HSV was detected in the respiratory tract were eligible for inclusion, regardless of study design, publication status or language. Information on our primary outcome (in-hospital all-cause mortality) and our secondary outcomes (30-day all-cause mortality, ICU all-cause mortality) must have been reported. There was no minimal number of patients. There were no minimal numbers of exclusion criteria. We searched *MEDLINE*, *ISI Web of Science* (*Science Citation Index Expanded*), *Cochrane Database* and ClinicalTrials.gov from inception to 25 May 2020 for eligible clinical studies. The search terms are provided (Supplementary Table 1). We complemented the database searches by screening the reference lists of relevant studies and reviews as well as by directly asking selected experts for studies that they were aware of but were not already included in this analysis. Two authors (S.W., S.H.) independently performed the literature search, identified all studies potentially relevant for this review and selected studies that were included. Conflicts over inclusion were resolved through consensus. All study authors were contacted in order to retrieve all additional available data (including information on missing data).

### Data extraction and risk of bias assessment

One author (S.H.) extracted the number of patients and events for both treatment groups (with or without antiviral therapy). A second author (S.W.) independently validated the results. One study [17] reported mortality; however, it did not specify the timepoint. We decided to include this article and classified it as hospital all-cause mortality. To assess potential heterogeneity of the study populations, we extracted information on study designs and settings as well as summarized patient characteristics. Two authors (S.W. and S.H.) independently performed a formal risk assessment of the individual studies according to the Newcastle-Ottawa Scale (NOS) (Supplementary Table 2) [18]. Differences in judgement were resolved by discussion. The certainty of evidence of the individual studies was judged according to the guidelines of the Grading of Recommendations Assessment, Development and Evaluation (GRADE) working group [19, 20].

### Synthesis of results

We applied random-effects meta-analyses to estimate relative risks (RRs) for the primary and secondary endpoints. Studies were pooled according to the Mantel-Haenszel and DerSimonian-Laird methods for within-study and between-study variance, respectively [21–23]. We applied a continuity correction of 0.5 in studies with cell frequencies of zero. Statistical heterogeneity was evaluated by the *I*^2^ statistic. Heterogeneity was judged accordingly: 0 to 40% = low, 30 to 60% = moderate, 50 to 90% = substantial (or high) and 75 to 100% = considerable. The importance of this measure depends on the magnitude and direction of effects as well as the precision of the estimate (often judged by the corresponding *p* value from the chi-squared test) [24]. To identify potential evidence of publication bias, we additionally inspected funnel plots. For the sensitivity analyses, we (i) performed the meta-analysis with odds ratios (ORs) as a measure of effect size, (ii) applied leave-one-out cross-validation and (iii) assumed that mortality reported in [17] was ICU all-cause mortality. We reported point estimates (RR or OR) together with their corresponding 95% confidence intervals (CIs) and presented the results as forest plots. All analyses were performed with R (version 3.6.0; R package meta, version 4.11.0) [25].

## Results

### Study selection

**Fig. 1:**
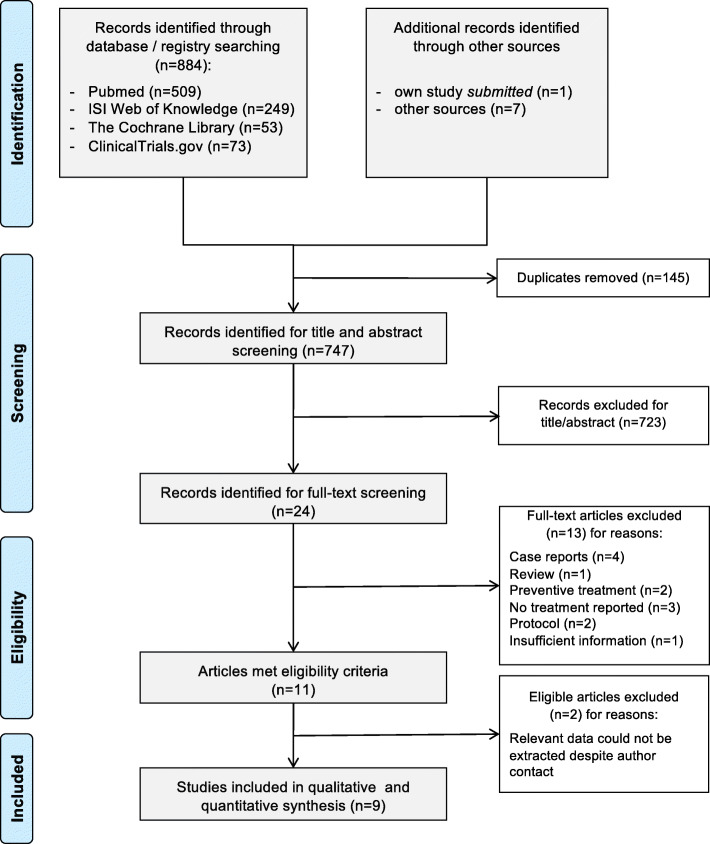
PRISMA flow diagram of study identification and selection process for outcome analysis

Our database search revealed 884 reports (see Table 1, Fig. 1). In addition, we considered one of our own studies, which was unpublished at that point [27], as well as seven studies from other sources. We removed 145 duplicates. Of the remaining 747 references, 738 studies were excluded due to lack of relevant information regarding our predefined outcome parameters. Finally, we identified and analysed nine studies comprising 1069 patients who had investigated at least one of the outcome parameters. All but one of these studies were non-randomized cohort studies, with the majority of retrospective design. Among the included studies, acyclovir was most often used for therapy. The only randomized study was performed by Luyt et al. [28]. In this double-blind, placebo-controlled trial, 238 patients who received mechanical ventilation for at least 96 h and continued to receive mechanical ventilation for at least 48 h with HSV oropharyngeal reactivation were included. The aim of the study was to determine whether pre-emptive treatment with intravenous acyclovir reduces the duration of mechanical ventilation in patients with HSV oropharyngeal reactivation. Detailed characteristics of the included studies are provided in Table 1.

**Table 1:** Characteristics of the included studies

Study	Design	Country	Patients (*n*)	Study period	Specimen and detection method	Clinical inclusion criteria	Immunosuppression	Primary endpoint	Secondary endpoints	Antiviral drug used (dosing), % of patients with therapy
Aisenberg et al. [26]	Single-centre, retrospective cohort study	USA	45	April 2000 to April 2004	BAL, viral culture alone or culture and cytology; HSV type not specified	Pneumonia	All patients with solid organ tumour	Hospital mortality	Median time on MV; median duration of ICU stay	Acyclovir (10 mg/kg tid), 68%; valacyclovir (1 g tid), 28%; famciclovir (500 mg tid), 4%
Camps et al. [17]	Single-centre, retrospective cohort study	Belgium	64	January 1992 to December 1997	BAL/TA, viral culture; HSV type not specified	Pneumonia	20%	Mortality	None	Acyclovir (5 mg tid 5 days),100%
Heimes et al. [27]	Single-centre, retrospective cohort study	Germany	306	January 2011 to December 2017	BAL/TA, PCR; HSV-1	Respiratory tract infection	34%	30-day mortality; survival	Hospital mortality; ICU mortality; length of hospital stay; length of ICU stay; duration of MV	Acyclovir (10 mg/kg tid 7 days), 91%; ganciclovir (no dose reported), 6%; both, 3%
Luyt et al. [7]	Single-centre, prospective cohort study	France	42	October 2004 to January 2006	BAL/TA, viral culture; HSV type not specified	Bronchopneumonitis in patients with prolonged MV (> 5 days)	Not specified	Hospital mortality	Length of ICU stay; duration MV; bacterial VAP	Acyclovir (10 mg/kg tid 5–14 days), 100%
Luyt et al. [28]	Double-blind, multicentre, placebo-controlled randomized clinical trial	France	238	February 2014 to February 2018	Oropharyngeal swab, PCR; HSV type not specified	MV for 96 h, predicted MV duration of ≥ 48 h and an HSV-positive oropharyngeal swab	Exclusion criteria	Ventilator-free days	60-day mortality; MV duration; occurrence of HSV bronchopneumonitis or active CMV infection; secondary bacterial pneumonia, bacteremia or fungemia; acute respiratory distress syndrome; septic shock post-randomization; acute renal failure	Acyclovir (5 mg/kg tid 14 days), 100%
Scheithauer et al. [5]	Single-centre, retrospective cohort study	Germany	51	January 2007 to April 2009	BAL/TA, PCR; HSV-1	Respiratory tract infection	Not specified	Hospital mortality	None	Acyclovir (no dose reported), 100%
Schuierer et al. [29, 30]	Single-centre, retrospective cohort study	Germany	89	January 2013 to April 2018	BAL/TA, PCR, HSV-1 and 2	Ventilator-associated pneumonia Exclusion criteria: neutronpenic patients	Steroids at baseline: 17–20%		Hospital mortality; ICU mortality; length of hospital stay; length of ICU stay; duration of MV	Acyclovir (9 mg/kg tid) [median, IQR 7–11]. Total acyclovir treatment duration of surviving patients was 10 days [median, IQR 6.5–14], 97%; ganciclovir (no dose reported), 3%
Traen et al. [31]	Single-centre, retrospective cohort study	Belgium	212	January 2004 to March 2012	BAL/TA, viral culture, HSV-1	Respiratory tract infection	9%	Hospital mortality; ICU mortality; length of ICU stay; total MV duration; need for vasopressors; need for inotropics; SOFA score	None	Acyclovir (10 mg/kg tid over the course of 5–14 days), 100%
van den Brink et al. [11]	Single-centre, retrospective cohort study	Netherlands	22	February 1996 to November 2001	BAL, viral culture, HSV-1	Pneumonia	Not specified	Not defined	None	Acyclovir (10 mg/kg tid), 95%; ganciclovir (5 mg/kg bd), 0%; both, 5%

*BAL* bronchoalveolar lavage, *CMV* Cytomegalovirus, *HSV* herpes simplex virus, *HSV-1* HSV type 1, *HSV-2* HSV type 2, *ICU* intensive care unit, *MV* mechanical ventilation, *PCR* polymerase chain reaction, *SOFA* sequential organ failure assessment, *TA* tracheal aspirate, *VAP* ventilator-associated pneumonia

### Hospital all-cause mortality

**Fig. 2:**
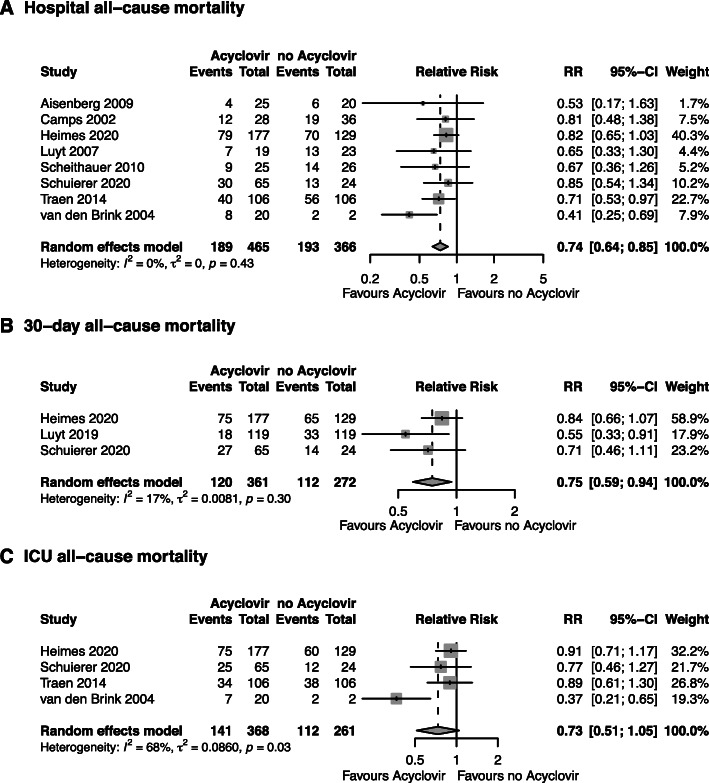
Results for the primary and secondary endpoints in mechanically ventilated patients with HSV detection in respiratory tract. CI, confidence interval; HSV, herpes simplex virus; ICU, intensive care unit; RR, relative risk

For the primary endpoint, hospital all-cause mortality, data from eight studies comprising 831 patients were used (Fig. 2a) [5, 7, 11, 17, 26, 27, 29, 31]. In these studies, hospital all-cause mortality was lower in patients with antiviral therapy (40.6%, 189 out of 465 patients) than in patients without antiviral therapy (52.7%, 193 out of 366 patients). This resulted in a RR of 0.74 [0.64, 0.85]. Heterogeneity was low in the studies reporting hospital all-cause mortality (*I*^2^ = 0%, *p* = 0.43). These results were supported by the sensitivity analyses (Supplementary Fig. 1A, Supplementary Table 3).

### Secondary outcomes

Overall, we identified three studies [27–29] reporting 30-day all-cause mortality in 633 patients (Fig. 2b) and four studies [11, 27, 29, 31] reporting ICU all-cause mortality in 629 patients (Fig. 2c). Pooling these studies, antiviral treatment was associated with lower 30-day all-cause mortality (RR 0.75 [0.59, 0.94]). However, no evidence for an association with ICU all-cause mortality was observed (RR 0.73 [0.51, 1.05]). Heterogeneity was low in 30-day all-cause mortality (*I*^2^ = 17%, *p* = 0.30) but substantial in ICU all-cause mortality (*I*^2^ = 68%, *p* = 0.03). These results were in line with those of the sensitivity analyses (Supplementary Fig. 1B and C, Supplementary Table 3; ICU all-cause mortality including [17]: RR 0.75 [0.57, 1.00], *I*^2^ = 56.5% with *p* = 0.06).

### Assessment of bias

Funnel plots revealed no evidence of publication bias, particularly for those studies reporting hospital all-cause mortality (Supplementary Fig. 2). However, the small number of studies reporting the secondary endpoints hampered the assessment. According to the GRADE classification, we judged the quality of evidence to be low for hospital all-cause mortality and very low for the secondary endpoints due to the inherent high risk of bias introduced by retrospective, non-randomized trial designs (Table 2). In particular, indication bias could not be ruled out, which could possibly over- or underestimate the favourable treatment effect of antiviral therapy (i.e. patients with a higher probability of survival are more likely to be treated and vice versa). Subcategories of bias (such as selection, allocation, performance, attrition or reporting bias) were not assessed.

**Table 2:** GRADE classification of main outcomes considering the different studies that contributed to the compiled effect estimate

Outcome	No. of participants (studies)	Risk of bias^1^	Inconsistency^2^	Indirectness	Imprecision^3^	Other considerations	Quality of the evidence (GRADE)	Events in acyclovir group	Events in control group	Relative risk (95% CI)	Anticipated absolute effects	
											Risk without acyclovir treatment	Risk difference with acyclovir treatment (95% CI)
Hospital all-cause mortality	831 (8)	Serious	Not serious	Not serious	Not serious	All plausible residual confounding would reduce the demonstrated effect	⨁⨁◯◯ Low	189/465 (40.6%)	193/366 (52.7%)	**0.74** (0.64; 0.85)	527 per 1.000	**137 fewer per 1.000** (from 190 fewer to 79 fewer)
30-day all-cause mortality	633 (3)	Serious	Not serious	Not serious	Serious	None	⨁◯◯◯ Very low	120/361 (33.2%)	112/272 (41.2%)	**0.75** (0.59; 0.94)	412 per 1.000	**103 fewer per 1.000** (from 169 fewer to 25 fewer)
ICU all-cause mortality	629 (4)	Serious	Not serious	Not serious	Serious	None	⨁◯◯◯ Very low	141/368 (38.3%)	112/261 (42.9%)	**0.73** (0.51; 1.05)	429 per 1.000	**116 fewer per 1.000** (from 210 fewer to 21 more)

*CI* confidence interval, *ICU* intensive care unit

^1^Risk of bias was high in all but one study due to the non-randomized study design

^2^Inconsistency (heterogeneity) was judged to be *not serious* when heterogeneity was low or moderate. Each issue judged as bearing a serious potential impact on the assessed features and rated as having a serious risk to the quality of evidence was downgraded by one level and, in the case of risk of bias, by two levels due to the high risk of bias

^3^Imprecision was assessed calculating the optimal information size (OIS) (*α* = 0.05; *β* = 0.1 and power 90%)

Link: https://gradepro.org

## Discussion

To the best of our knowledge, this is the first systematic review and meta-analysis aiming to summarize the current evidence for antiviral therapy for mechanically ventilated ICU patients in whom HSV was detected in the respiratory tract. Our literature search identified nine studies with 1069 patients overall. The results of the meta-analysis of the available data showed that antiviral therapy might improve hospital all-cause mortality as well as 30-day all-cause mortality in mechanically ventilated patients in whom HSV was detected in the respiratory tract. These results suggest that the detection of HSV in these circumstances is of clinical relevance, albeit most likely only in some cases, not all. This is supported by an observation from Luyt et al*.* [7]. In that retrospective study, in 32.6% of 129 patients in whom HSV was detected in the BAL, HSV bronchopneumonitis was histologically confirmed. However, due to its complexity and risks, a lung biopsy for securing a diagnosis of HSV bronchopneumonitis is not routinely feasible in critically ill patients. In addition, cytologic changes typical for HSV infection, i.e. multinucleated giant cells with specific nuclear inclusions, are admittedly specific but suffer from poor sensitivity [32]. On the other hand, rendering a confident clinical diagnosis of HSV bronchopulmonary infection in mechanically ventilated patients without lung biopsy is challenging. Clinical symptoms of HSV bronchopneumonitis are nonspecific and often mimic bacterial pneumonia, with fever, hypoxemia and purulent pulmonary secretions. The same is true for the radiologic examinations, which are often nonspecific and can show ground-glass attenuations, air-space consolidations and interlobular thickening [16].

Randomized controlled studies are necessary to identify patients who could benefit (the most) from antiviral therapy and to reduce therapy-associated adverse events in others. In addition to clinical signs of a respiratory tract infection, possible criteria might be location of detection, i.e. upper respiratory tract/oropharyngeal cavity versus lower respiratory tract/BAL [31] and amount of virus load [29]. For example, Traen et al. [31] retrospectively analysed 212 ICU patients with a positive HSV-1 culture from the endotracheal/bronchial aspirate (*n* = 162) or BAL (*n* = 50). In their study, using propensity score matching, acyclovir therapy was associated with lower ICU mortality (OR 0.31, 95% CI 0.18–0.56) and lower in-hospital mortality (OR 0.28, 95% CI 0.17–0.46). In particular, the subgroup of patients with HSV-1 detected in the BAL accounted mostly for this difference. Most recently, in a retrospective study, Schuierer et al. [29] investigated whether patients with ventilator-associated pneumonia (VAP) not responding to antibiotics and in whom HSV could be detected in respiratory secretions (BAL or tracheal aspirates) would benefit from acyclovir treatment. In their cohort of 425 patients screened for HSV type 1 or 2, 57 (13.4%) patients had a low viral load (10^3^–10^5^ HSV copies/ml) and 69 (16.2%) patients a high (> 10^5^ HSV copies/ml) viral load. Thirty patients (7%) with a low viral load and 59 (14%) patients with a high viral load fulfilled the strict inclusion criteria, i.e. VAP not responding to antibiotics, and were included in the analyses. The authors observed in patients with a high viral load that acyclovir therapy was associated with lower hazard rates for ICU death (treated, 20 out 49 patients died; untreated, 6 out of 10 died; hazard ratio (HR) 0.31, 95% CI 0.11–0.92) as well as for 30-day mortality (treated, 21 out of 49 patients died; untreated, 8 out of 10; HR 0.32, 95% CI 0.12–0.85) and resulted in better circulatory and pulmonary oxygenation function over the course of acyclovir treatment compared to no acyclovir treatment [29, 30]. Thus, material from the lower respiratory tract and evaluation of viral load in combination with clinical signs and high likelihood of viral pneumonia might be helpful for identifying patients who may benefit the most from antiviral therapy. However, the possibility of adverse events related to antiviral therapy has also to be taken into account. Nephrotoxicity is the most important side effect of acyclovir, with an overall incidence of acute kidney injury (AKI) of 13%, half of which are KDIGO grade 2/3, as recently reported by Ryan et al. [33]. However, studies showed that acyclovir-associated nephrotoxicity was usually reversible and could be minimized by slow infusion and adequate hydration [34]. Moreover, three of the studies included in this meta-analysis reporting nephrotoxicity did not observe a significant deterioration of renal function [27–30].

### Limitations

The findings and interpretations of this meta-analysis and systematic review are limited by the quality of available evidence. The majority of available evidence was derived from non-randomized, single-centre studies with an inherent high risk of bias. In addition, studies were heterogeneous in terms of included and analysed study cohorts, ranging from patients with HSV detection in the oropharyngeal cavity only [28] to patients with histologically confirmed HSV bronchopneumonitis [7]. To account for this heterogeneity, we used random-effects meta-analyses and performed sensitivity analyses that supported the results of our main analyses. Finally, we did not manually search for unpublished studies, other than conference proceedings that are covered by the utilized electronic databases. Notwithstanding these limitations, the present meta-analysis provides the most comprehensive evaluation of the evidence for antiviral therapy for mechanically ventilated ICU patients in whom HSV was detected in the respiratory tract.

## Conclusion

HSV is frequently detected in the respiratory tract of mechanically ventilated ICU patients. Our meta-analysis of the available data suggests that antiviral therapy is associated with lower hospital all-cause mortality as well as 30-day all-cause mortality. Multicentre, randomized controlled studies are urgently required to identify patients who may benefit the most from antiviral therapy.

## Supplementary information

**Additional file 1: Supplementary Table 1.** Literature search terms. **Supplementary Table 2.** Newcastle-Ottawa Scale (NOS) assessing the quality of the individual studies included in the meta-analysis. **Footnotes:** ^1^ The main outcome was in-hospital overall mortality. ^2^ Only a few were patients included. Comparability cannot be assessed. *Selection.* 1) Representativeness of the exposed cohort: a) truly representative of intensive care patients*, b) somewhat representative of the average intensive care patient *, c) selective group of patients at the ICU only, d) no description of the derivation of the cohort. 2) Selection of the non-exposed cohort: a) drawn from the same population as the exposed cohort *, b) drawn from a different source, c) no description of the derivation of the non-exposed cohort. 3) Ascertainment of exposure: a) secure record and rated as appropriate if diagnosis was based on PCR results or culture from bronchoalveolar lavage or swaps* b) structured interview, c) written self-report, d) no descriptio. 4) Demonstration that outcome of interest was not present at start of study: a) yes *, b) no. *Comparabilit.* 1) Comparability of cohorts on the basis of the design or analysis; a) study controls for the most important factor, such as age, gender, comorbidities *, b) ** Rated as appropriate with ** when propensity score matching or a prospective randomized trial was performed. *Outcome.* 1) Assessment of outcome; a) independent blind assessment *, b) record linkage *, c) self-reported, d) no descriptio. 2) Was follow-up long enough for outcomes to occur? a) yes (in hospital mortality) *, b) no. 3) Adequacy of follow up of cohorts, a) complete follow up - all subjects accounted for *, b) subjects lost to follow up unlikely to introduce bias - small number lost - > 20% *, c) Kaplan-Meier Plot Curve provided *, d) follow up rate < 20% and no description of those lost, e) no statement. **Supplementary Table 3** Results of leave-one-out cross-validation meta-analyses for the primary and secondary endpoints in herpes simplex virus (HSV) patients, comparing those who were treated with acyclovir to those who were not treated with antiviral drug. The pooled relative risk estimate (RR) and its 95% confidence interval (CI) as well as the heterogeneity statistic I^2^ were considered when omitting the indicated study. Abbreviations: ICU, intensive care unit.

**Additional file 2: Supplementary Figure 1.** Results for the primary and the secondary endpoints in mechanically ventilated patients with HSV detection in respiratory tract (measure of effect size: odds ratio). Abbreviations: CI, confidence interval; HSV, herpes simplex virus; ICU, intensive care unit; OR, odds ratio.

**Additional file 3: Supplementary Figure 2.** Assessment of publication bias for the primary and secondary endpoints. In the funnel plot, the individual study results are represented as grey points, and the pooled estimate is indicated by a dotted line. Abbreviations: ICU, intensive care unit.

## Data Availability

All data generated or analysed during this study are included in this published article.
