# Supplementary material for: Effect of antiviral therapy on the outcomes of mechanically ventilated patients with herpes simplex virus detected in the respiratory tract: a systematic review and meta-analysis
Source: Crit Care. 2020 Sep 29;24:584. doi: 10.1186/s13054-020-03296-5 (PMC7522924; doi:10.1186/s13054-020-03296-5)
Supplement: Supplementary file 1 — Additional file 1: Supplementary Table 1. Literature search terms. Supplementary Table 2. Newcastle-Ottawa Scale (NOS) assessing the quality of the individual studies included in the meta-analysis. Footnotes: 1 The main outcome was in-hospital overall mortality. 2 Only a few were patients included. Comparability cannot be assessed. Selection. 1) Representativeness of the exposed cohort: a) truly representative of intensive care patients*, b) somewhat representative of the average intensive care patient *, c) selective group of patients at the ICU only, d) no description of the derivation of the cohort. 2) Selection of the non-exposed cohort: a) drawn from the same population as the exposed cohort *, b) drawn from a different source, c) no description of the derivation of the non-exposed cohort. 3) Ascertainment of exposure: a) secure record and rated as appropriate if diagnosis was based on PCR results or culture from bronchoalveolar lavage or swaps* b) structured interview, c) written self-report, d) no descriptio. 4) Demonstration that outcome of interest was not present at start of study: a) yes *, b) no. Comparabilit. 1) Comparability of cohorts on the basis of the design or analysis; a) study controls for the most important factor, such as age, gender, comorbidities *, b) ** Rated as appropriate with ** when propensity score matching or a prospective randomized trial was performed. Outcome. 1) Assessment of outcome; a) independent blind assessment *, b) record linkage *, c) self-reported, d) no descriptio. 2) Was follow-up long enough for outcomes to occur? a) yes (in hospital mortality) *, b) no. 3) Adequacy of follow up of cohorts, a) complete follow up - all subjects accounted for *, b) subjects lost to follow up unlikely to introduce bias - small number lost - > 20% *, c) Kaplan-Meier Plot Curve provided *, d) follow up rate < 20% and no description of those lost, e) no statement. Supplementary Table 3 Results of leave-one-out cross-validation meta-analyses [file 13054_2020_3296_MOESM1_ESM.docx]

**Supplementary Tables**

**Supplementary Table 1.** Literature search terms

| **Database** | **Search Terms** | **Hits** |
| --- | --- | --- |
| **Pubmed** | (((((study OR trial OR case report OR case series OR Clinical Study [MeSH Terms] OR random* OR retrospec* OR prospec* OR cohort))) AND ((aciclovir OR acyclovir OR valaciclovir OR valchlor OR valacyclovir OR valaciclovir OR valacyclovir OR famciclovir OR famciclovir))) AND ((Lung OR pulmonary OR (acute AND respiratory AND distress AND syndrome) OR (adult AND respiratory AND distress AND syndrome) OR adult respiratory distress syndrome OR HAP OR CAP OR pleuropneumon* OR bronchopneumon* OR pneumon* OR pneumonia OR pneumonia [MeSH Terms] OR bronchoalveolar lavage [MeSH Terms] OR oropharyng* OR tracheo* OR Ventilate* OR Mechanic* OR lavage))) AND ((HSV OR Herpes simplex OR Herpes simplex [MeSH Terms] OR Herpe* OR simplex OR herpetic)) | 509 |
| **ISI Web of Science** | (((((study OR trial OR case report OR case series OR Clinical Study [MeSH Terms] OR random* OR retrospec* OR prospec* OR cohort))) AND ((aciclovir OR acyclovir OR valaciclovir OR valchlor OR valacyclovir OR valaciclovir OR valacyclovir OR famciclovir OR famciclovir))) AND ((Lung OR pulmonary OR (acute AND respiratory AND distress AND syndrome) OR (adult AND respiratory AND distress AND syndrome) OR adult respiratory distress syndrome OR HAP OR CAP OR pleuropneumon* OR bronchopneumon* OR pneumon* OR pneumonia OR pneumonia [MeSH Terms] OR bronchoalveolar lavage [MeSH Terms] OR oropharyng* OR tracheo* OR Ventilate* OR Mechanic* OR lavage))) AND ((HSV OR Herpes simplex OR Herpes simplex [MeSH Terms] OR Herpe* OR simplex OR herpetic)) | 249 |
| **Cochrane Database of Systematic Reviews** | (HSV OR Herpes OR Herpes simplex OR simplex OR herpetic) AND (aciclovir OR acyclovir OR valaciclovir OR valchlor OR valacyclovir OR valaciclovir OR valacyclovir OR famciclovir OR famciclovir) AND (Lung OR pulmonary OR (acute AND respiratory AND distress AND syndrome) OR (adult AND respiratory AND distress AND syndrome) OR adult respiratory distress syndrome OR HAP OR CAP OR pleuropneumonia OR bronchopneumonia OR pneumonia OR oropharyngeal OR tracheo OR Ventilation OR Mechanic OR lavage) AND (study OR trial OR case report OR case series OR Clinical Study OR randomized OR retrospective OR prospective OR cohort) | 53 |
| **Clinical trials.gov** | (Acyclovir OR aciclovir) AND (HSV OR Herpes Simplex OR Herpesvirus) and status (completed OR terminated) | 73 |

**Supplementary Table 2.** Newcastle-Ottawa Scale (NOS) assessing the quality of the individual studies included in the meta-analysis.

| **Study** | **Selection**  **(maximum ****)** | | | | **Comparability (maximum **)** | **Outcome** | | | **Total Score**  **(maximum 9)** |
| --- | --- | --- | --- | --- | --- | --- | --- | --- | --- |
|  | Representativeness of the exposed cohort | Selection of the non-exposed cohort | Ascertainment of exposure | Demonstration that outcome of interest was not present at start of study^1^ | Comparability of cohorts on the basis of the design or analysis | Assessment of outcome | Was follow-up long enough for outcomes to occur? | Adequacy of follow up of cohorts |  |
| **Aisenberg *et al. 2009*** | ***** | ***** | ***** | ***** | ***** | ***** | ***** |  | **7** |
| **Camps *et al.* 2002** |  |  | ***** | ***** |  | ***** | ***** |  | **4** |
| **Heimes *et al.* 2020** | ***** | ***** | ***** | ***** | ***** | ***** | ***** | ***** | **8** |
| **Luyt *et al.* 2007** | ***** | ***** | ***** | ***** | ***** | ***** | ***** |  | **7** |
| **Luyt *et al.* 2019** | ***** | ***** | ***** | ***** | ****** | ***** | ***** | ***** | **9** |
| **Scheithauer *et al.* 2010** |  | ***** | ***** | ***** | ***** | ***** | ***** |  | **6** |
| **Schuierer *et al.* 2020** | ***** | ***** | ***** | ***** | ***** | ***** | ***** | ***** | **7** |
| **Traen *et al.***  **2014** | ***** | ***** | ***** | ***** | ****** | ***** | ***** | ***** | **9** |
| **Van den Brink *et al.* 2004** ^2^ |  |  | ***** | ***** |  | ***** | ***** |  | **4** |

**Footnotes:**

^1^ The main outcome was in-hospital overall mortality. ^2^ Only a few were patients included. Comparability cannot be assessed.

*Selection*

1) Representativeness of the exposed cohort: a) truly representative of intensive care patients*****, b) somewhat representative of the average intensive care patient *****, c) selective group of patients at the ICU only, d) no description of the derivation of the cohort

2) Selection of the non-exposed cohort: a) drawn from the same population as the exposed cohort *****, b) drawn from a different source, c) no description of the derivation of the non-exposed cohort

3) Ascertainment of exposure: a) secure record and rated as appropriate if diagnosis was based on PCR results or culture from bronchoalveolar lavage or swaps* b) structured interview, c) written self-report, d) no description

4) Demonstration that outcome of interest was not present at start of study: a) yes ***,** b) no

*Comparability*

1) Comparability of cohorts on the basis of the design or analysis; a) study controls for the most important factor, such as age, gender, comorbidities *****, b) ****** Rated as appropriate with ** when propensity score matching or a prospective randomized trial was performed.

*Outcome*

1) Assessment of outcome; a) independent blind assessment *****, b) record linkage *****, c) self-reported, d) no description

2) Was follow-up long enough for outcomes to occur? a) yes (in hospital mortality) *****, b) no

3) Adequacy of follow up of cohorts, a) complete follow up - all subjects accounted for *****, b) subjects lost to follow up unlikely to introduce bias - small number lost - > 20% *****, c) Kaplan-Meier Plot Curve provided *****, d) follow up rate < 20% and no description of those lost, e) no statement

**Supplementary Table 3.** Results of leave-one-out cross-validation meta-analyses for the primary and secondary endpoints in herpes simplex virus (HSV) patients, comparing those who were treated with acyclovir to those who were not treated with antiviral drug. The pooled relative risk estimate (RR) and its 95% confidence interval (CI) as well as the heterogeneity statistic I^2^ were considered when omitting the indicated study. Abbreviations: ICU, intensive care unit.

| Omitted study | RR [95% CI] | I^2^ [%] |
| --- | --- | --- |
| *Hospital all-cause mortality* | | |
| Aisenberg 2009 | 0.73 [0.62, 0.86] | 10.6 |
| Camps 2002 | 0.72 [0.60, 0.85] | 12.3 |
| Heimes 2020 | 0.68 [0.57, 0.82] | 0.0 |
| Luyt 2007 | 0.73 [0.62, 0.86] | 12.9 |
| Scheithauer 2010 | 0.73 [0.61, 0.86] | 13.3 |
| Schuierer 2020 | 0.71 [0.60, 0.84] | 8.5 |
| Traen 2014 | 0.72 [0.59, 0.88] | 14.7 |
| van den Brink 2004 | 0.77 [0.66, 0.90] | 0.0 |
| *ICU all-cause mortality* | | |
| Heimes 2020 | 0.65 [0.37, 1.13] | 75.2 |
| Schuierer 2020 | 0.71 [0.44, 1.14] | 78.5 |
| Traen 2014 | 0.66 [0.39, 1.13] | 77.9 |
| van den Brink 2004 | 0.88 [0.73, 1.07] | 0.0 |
| *30-day all-cause mortality* | | |
| Heimes 2020 | 0.64 [0.45, 0.89] | 0.0 |
| Luyt 2019 | 0.81 [0.65, 1.00] | 0.0 |
| Schuierer 2020 | 0.72 [0.47, 1.09] | 56.9 |
