# Supplementary figures and images for: Effect of antiviral therapy on the outcomes of mechanically ventilated patients with herpes simplex virus detected in the respiratory tract: a systematic review and meta-analysis
Source: Crit Care. 2020 Sep 29;24:584. doi: 10.1186/s13054-020-03296-5 (PMC7522924; doi:10.1186/s13054-020-03296-5)

**A Hospital all-cause mortality**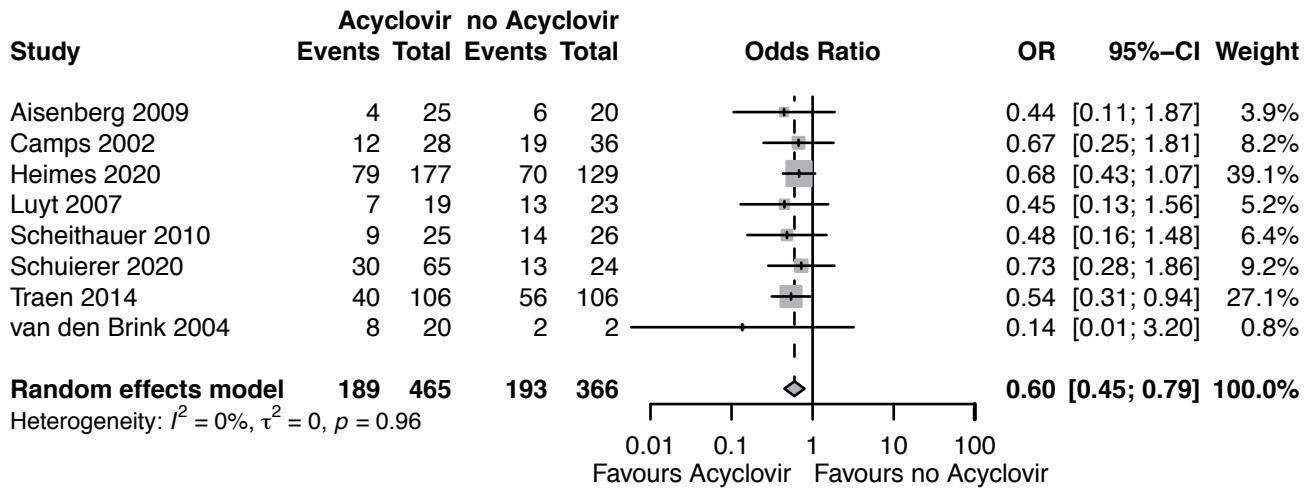**B 30-day all-cause mortality**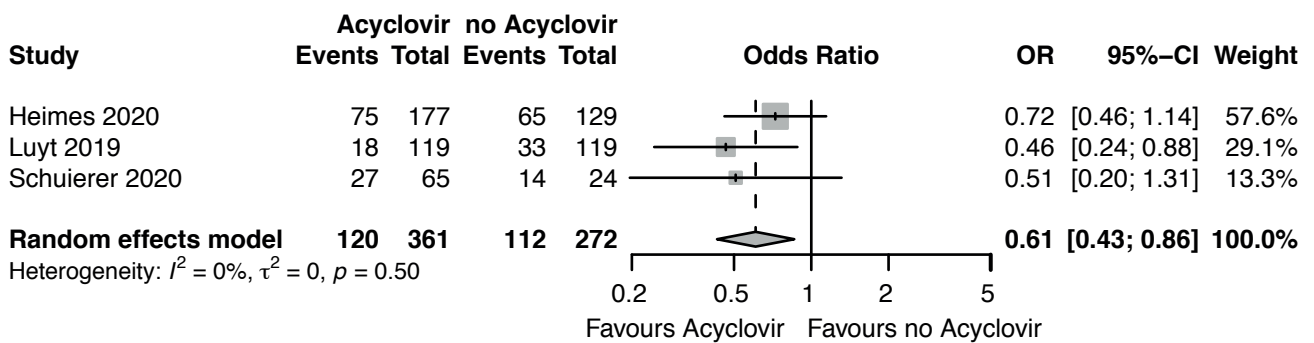**C ICU all-cause mortality**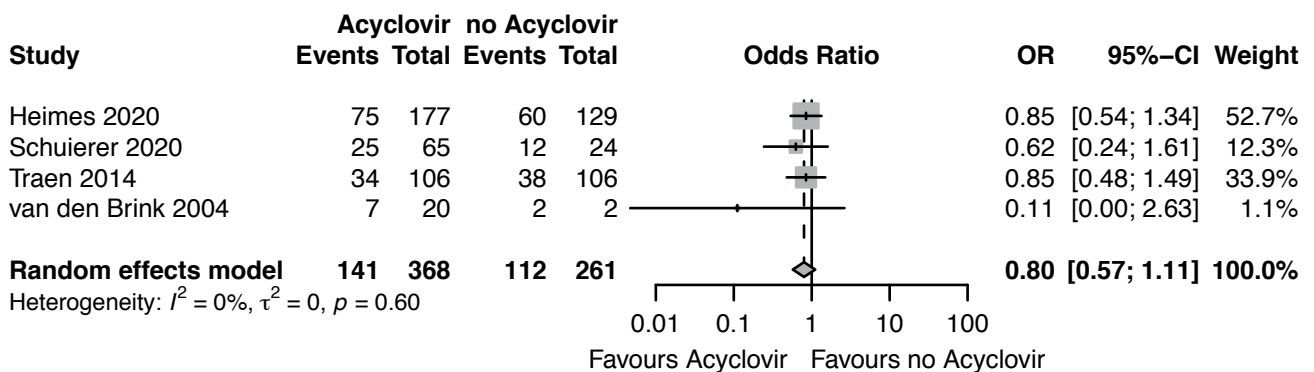

Supplement: Supplementary file 2 — Additional file 2: Supplementary Figure 1. Results for the primary and the secondary endpoints in mechanically ventilated patients with HSV detection in respiratory tract (measure of effect size: odds ratio). Abbreviations: CI, confidence interval; HSV, herpes simplex virus; ICU, intensive care unit; OR, odds ratio. [file 13054_2020_3296_MOESM2_ESM.pdf]

**A Hospital all-cause mortality**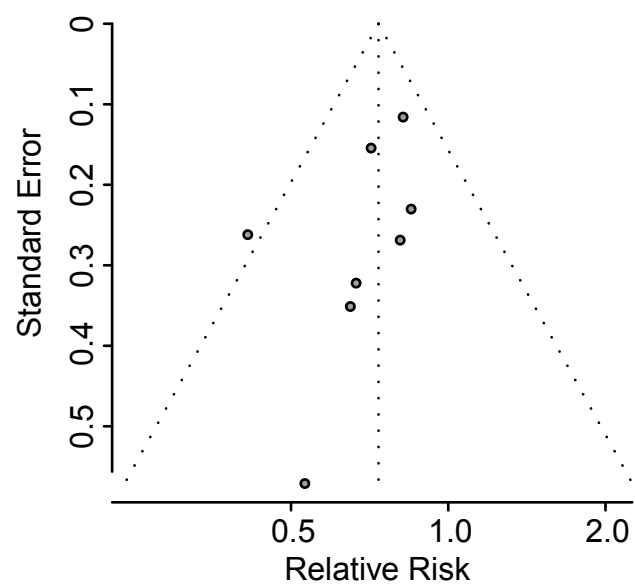**B 30-day all-cause mortality**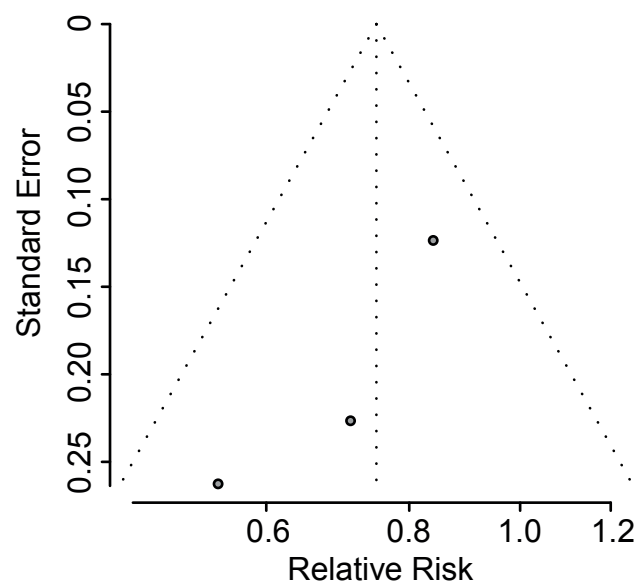**C ICU all-cause mortality**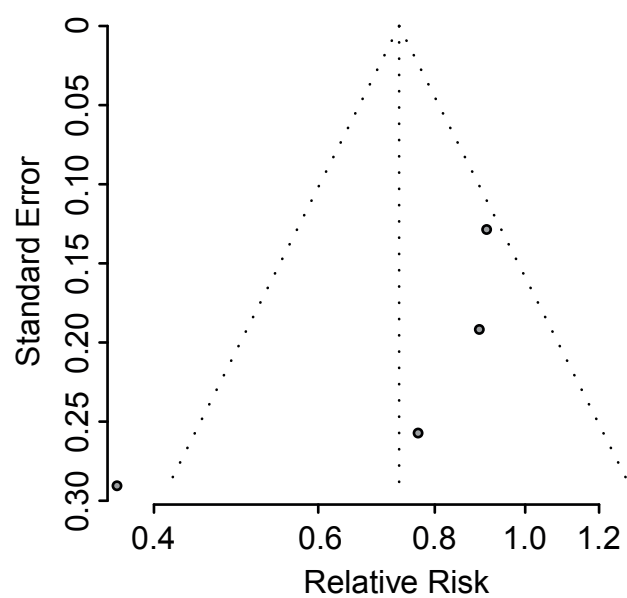

Supplement: Supplementary file 3 — Additional file 3: Supplementary Figure 2. Assessment of publication bias for the primary and secondary endpoints. In the funnel plot, the individual study results are represented as grey points, and the pooled estimate is indicated by a dotted line. Abbreviations: ICU, intensive care unit. [file 13054_2020_3296_MOESM3_ESM.pdf]
